# Case Report: A Case of Acute Cellular Rejection Due to Atopic Dermatitis Exacerbation 3 Years After Heart Transplantation

**DOI:** 10.3389/fimmu.2021.630051

**Published:** 2021-02-22

**Authors:** Nobutaka Kakuda, Eisuke Amiya, Masaru Hatano, Hisataka Maki, Chie Bujo, Masaki Tsuji, Koichi Narita, Kanna Fujita, Junichi Ishida, Minoru Ono, Issei Komuro

**Affiliations:** ^1^Department of Cardiovascular Medicine, Graduate School of Medicine, The University of Tokyo, Tokyo, Japan; ^2^Department of Therapeutic Strategy for Heart Failure, The University of Tokyo, Tokyo, Japan; ^3^Department of Cardiovascular Medicine, Saitama Medical Center, Jichi Medical University, Saitama, Japan; ^4^Department of Cardiac Surgery, Graduate School of Medicine, The University of Tokyo, Tokyo, Japan

**Keywords:** atopic dermatitis, regulatory T cell, acute cellular rejection, heart transplantation, late rejection of graft

## Abstract

**Background:**

Little evidence has been presented about the association between previous atopic/allergic disease and graft rejection after solid organ transplantation. Thus, we present a case wherein acute cellular rejection (ACR) after heart transplantation (HTx) was noted along with exacerbation of atopic disease.

**Case Summary:**

A 32-year-old man was admitted at our hospital for regular monitoring of graft rejection. He had undergone heart transplant 3 years prior due to dilated cardiomyopathy. Echocardiogram revealed good biventricular function, and no abnormal findings were found in blood sampling tests. However, biopsy showed moderate ACR [Grade 2R(ISHLT 2004)/3A(ISHLT 1990)], which required twice-repeated steroid pulses with intensified immunosuppression. Meanwhile, his atopic dermatitis, which was diagnosed before having heart failure, was getting worse for the past 6 months. The exacerbation of atopic dermatitis was presumed to be related to the development of the intractable cellular rejection.

**Discussion:**

This case suggested the association of atopic disease and graft rejection after HTx. We examined 76 patients from a cohort of previous studies who underwent HTx at our hospital, which suggested that patients with atopic/allergic disorders such as atopic dermatitis and asthma tended to have a significantly higher frequency of moderate rejection than non-allergic patients. (p = 0.012; Fisher’s exact test). Our case also suggests that exacerbation of atopic dermatitis might cause graft rejection of the transplanted organ, so that it is important to carefully evaluate the risk of graft rejection if there is a previous history of atopic/allergic disease.

## Introduction

Heart transplantation (HTx) is a radical treatment that saves the lives of those with end-stage heart failure. Although the surgical procedures and perioperative management methods have been established, control of acute and chronic rejection and the harmful effects of immunosuppressants remain as the biggest challenges. Previous studies have reported that through the response of the T-cells, the frequency of graft rejection increases in the presence of atopic/allergic diseases (1, 2). The pathophysiology of many atopic/allergic diseases is associated with a type 2 T helpler cell (Th2)-based inflammatory response that involves the production of cytokines such as interleukin (IL)-4, IL-5, and IL-13 (3). The untoward effect of Th2-based inflammation and cytokines is presumed to increase graft rejection by making CD4^+^ effector T-cells resistant to regulatory T-cells (Tregs) (4). Several studies have also reported the critical role Tregs play in immune tolerance and immunosuppression (5, 6). In this report, we present a case of graft rejection reaction that correlated with aggravation of atopic dermatitis 3 years after HTx.

## Case Description

**Figure 1:**
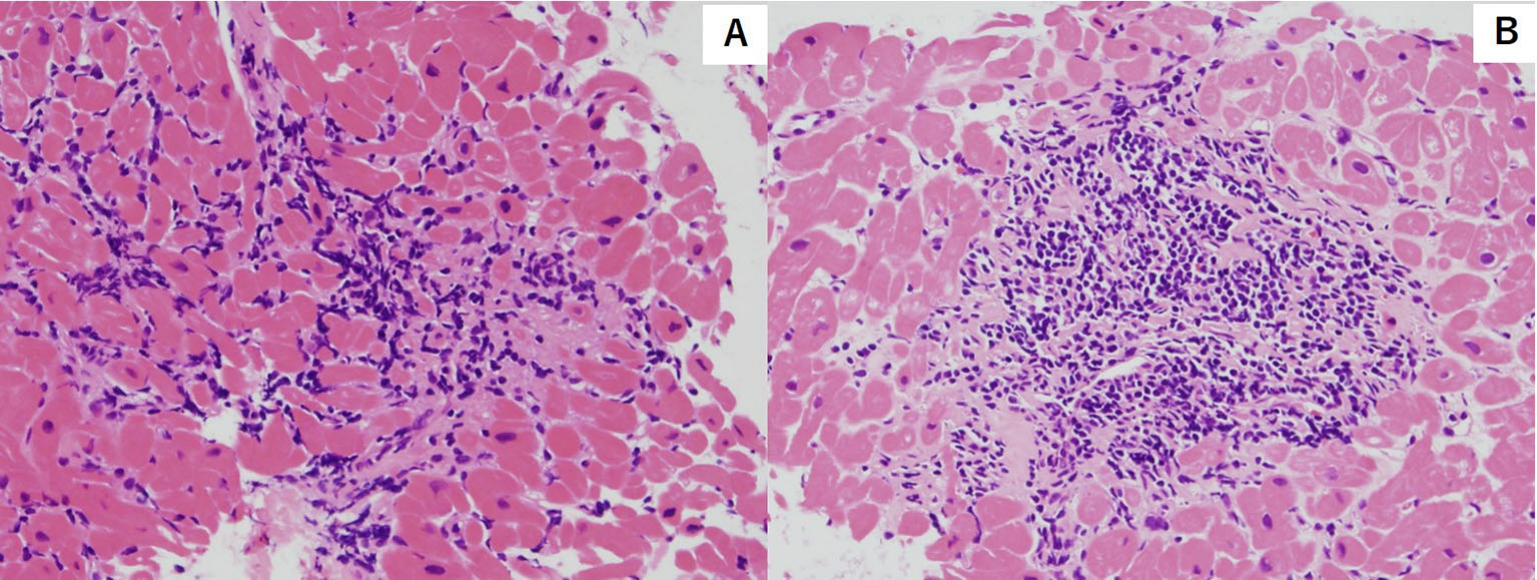
Hematoxylin and eosin stain of endocardial biopsy sample featuring myocyte injury with multiple lymphocytic infiltration. [Grade2R(ISHLT 2004)/3A(ISHLT 1990)]. **(A)** First endocardial biopsy **(B)** Second endocardial biopsy.

We present the case of a 32-year-old man who underwent orthotropic HTx at our hospital due to dilated cardiomyopathy (DCM). The patient was diagnosed with atopic dermatitis during childhood which necessitated the use of topical drugs. At 24 years old, he noted dyspnea on exertion. Shortly after, he was diagnosed with DCM and subsequently developed severe drug-resistant heart failure. At 25 years old, a left ventricular assist device was implanted as bridge therapy for heart failure. At 29 years old, orthotropic HTx was performed at our hospital. The patient was treated with standard immunosuppression therapy consisting of cyclosporine, mycophenolate mofetil (MMF), and prednisolone. We performed endomyocardial biopsy according to our institutional protocol, which consists of examinations at the 1^st^, 2^nd^, 3^rd^, 4^th^, 6^th^, 8^th^, 10^th^, 12^th^, 18^th^, and 24^th^ post-procedural weeks and the 1^st^, and 2^nd^ post-procedural years. During the patient’s follow-ups, there were three events of mild acute cellular rejection (ACR) (grade 1R ISHLT2004/grade 2 ISHLT 1990) within two years, whereas in all other examinations there were no findings of ACR. In addition, anti-HLA antibodies were not detected during any of the follow-ups. There were also no signs of heart failure such as dyspnea and edema, and the immunosuppressive treatment course was stable. The dose of prednisolone was gradually reduced according to the policy of our institution and it was turned off about one year after HTx. After 3 years, the patient was hospitalized to undergo a regular endomyocardial biopsy (EMB). On admission, his vital signs were normal, with no leg edema and no jugular dilation. Of note is that his atopic dermatitis worsened from the previous winter season and six months before hospitalization. This was evidenced by erythema and scaling on the neck, precordium, and back, and erythema with scabs and exudate were found on the arms and legs. A blood test on admission revealed mild anemia. The blood counts of the other two strains were within the normal range, and the eosinophil count was normal. Liver and kidney function tests were normal. Chest X-ray showed normal heart shadow and no pleural effusion. Electrocardiogram (ECG) revealed sinus rhythm of 73 bpm with no ST-T changes. Transthoracic echocardiography showed normal left and right ventricular function, and left ventricle ejection fraction was estimated at 73% (Teichholz). Echocardiography parameters of intraventricular and posterior left ventricle wall diameters were 6 and 8 mm, respectively. The immunosuppressive drugs taken by the patient at the time of hospitalization were MMF 1,750 mg/day and cyclosporine 150 mg/day. Cyclosporine serum level (the trough value) was 145 ng/ml, which was within the optimum range. Right heart catheterization showed that the mean right atrial pressure, mean pulmonary artery wedge pressure, mean pulmonary artery pressure, and cardiac index (Fick) were 5, 11, 16 mmHg, and 3.63 L/min/m^2^, respectively, suggesting that intracardiac pressure was within the normal range and cardiac output was maintained. Coronary artery angiography showed no significant stenosis in the coronary arteries. On the other hand, EMB showed infiltration of multiple inflammatory cells with myocardial damage, which corresponded ACR with grade 2R (ISHLT 2004)/grade (3A ISHLT 1990) (**Figure 1**). To counteract the graft rejection, he was treated with a 3-day course of parenteral methylprednisolone (1,000 mg/day) and then oral prednisolone 25 mg/day, which was gradually reduced every two days. In addition, the dose of cyclosporine was increased from 150 mg/day to 160 mg/day. However, repeat EMB after 1 week also revealed similar findings of ACR with a grade of 2R (ISHLT 2004)/3A (ISHLT 1990). The patient was given steroid pulse therapy for 3 days, and MMF was shifted to everolimus 1 mg/day to intensify immunosuppression. Repeat EMB after 1 week showed the improvement of graft rejection (Grade 0). He was discharged without any overt complications. In addition, atopic dermatitis also improved by intensifying immunosuppressive drugs with topical drugs. During subsequent follow-ups, no adverse or clinical events were observed. One year after, 4 years after HTx, significant ACR was not noted [grade 1R (ISHLT 2004)/1A (ISHLT 1990)] and there was no exacerbation of atopic dermatitis.

## Discussion

Previous studies have reported cases in which concomitant atopic/allergic disorders had an increased incidence of graft rejection. Previous investigations using mice have demonstrated that classic allergic disease such as airway hyperresponsiveness and allergic conjunctivitis, exacerbates corneal allograft rejection (1, 4). Nguyen et al. have indicated that the frequency of corneal graft rejection following normal-risk keratoplasty was significantly increased in patients with atopic dermatitis (2). Seung et al. have shown that acute rejection after renal transplantation is more common and severe in patients with atopy (7). However, no case reports exist about the association of atopic/allergic disease and graft rejection after HTx, and in our knowledge, this is the first report of its kind.

Several hypotheses have been proposed to explain the association between atopic/allergic disease and graft rejection. Atopic dermatitis and allergic airway inflammation are Th2-dominant allergic diseases. Th2 produces cytokines such as IL-4, which induces immunoglobulin E (IgE) production by acting on B-cells, while mast cells release cytokines such as IL-6 and tumor growth factor-beta (TGF-β) in response to the involvement of the IgE receptor (FcϵRI) complex on the cell surface (3, 8). The cytokines induced by Th2 are reported to suppress the effect of Tregs, leading to the enhancement of CD4^+^ effector T-cells. In studies with mice, it has been reported that IL-4 exacerbates corneal allograft rejection by making CD4^+^ effector T-cells resistant to Tregs (9). Tregs have been reported to play important roles in the suppression of graft rejection following organ transplant (3). From these findings, there is a possibility that the risk of rejection may increase *via* such T-cell responses in patients with atopic/allergic diseases. On the other hand, several reports have described the association between eosinophil and graft rejection after heart and lung transplantation (10–12). Acute graft rejection was reported to be associated with the local productions of IL-4 and IL-5 together with eosinophil infiltration (10). Several studies have reported on the impact of eosinophil on the development of graft rejection (11, 12). Eosinophil was reported to correlate with the severity of atopic dermatitis (13), and thus, might add some hints for the explanation of the association between atopy and graft rejection. However, the present case did not represent eosinophilia, so the correct explanation of the association between atopic dermatitis and graft rejection in this case had not been clarified.

**Figure 2:**
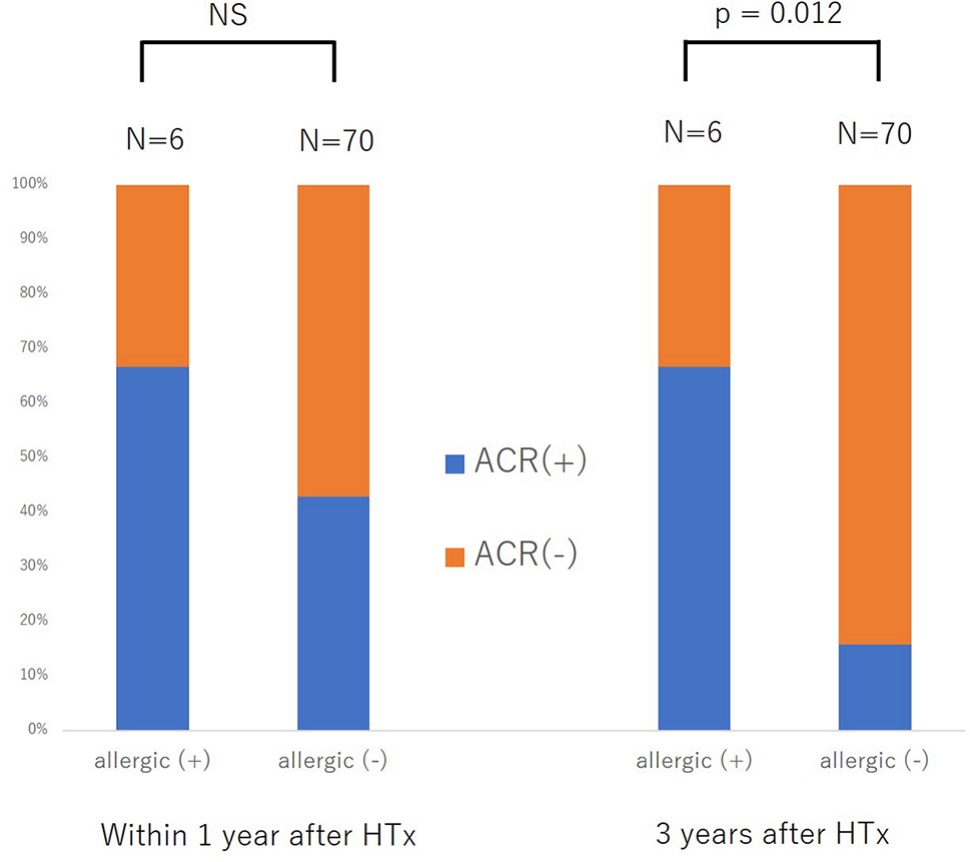
Difference in the development of acute cellular rejection within one year after heart transplantation (HTx) and three years after HTx between patients with and without previous allergic disease. NS, not significant.

**Figure 3:**
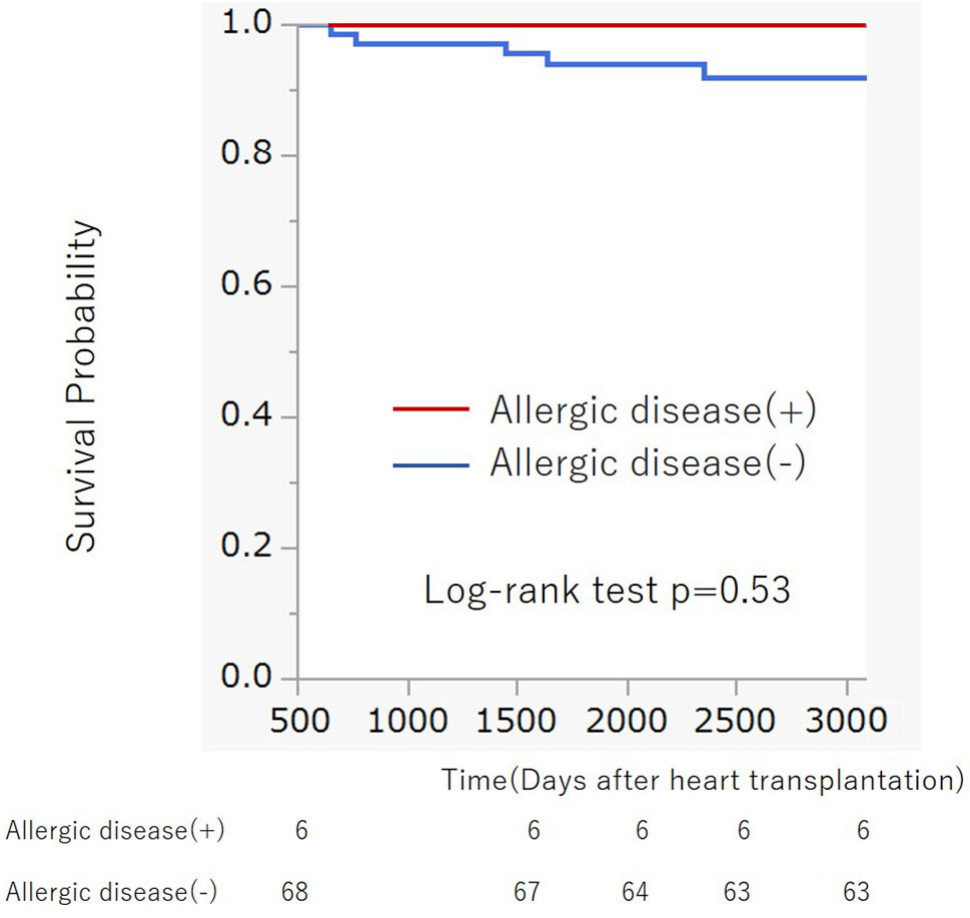
Kaplan-Meier survival curve of patients with and without previous allergic disease after heart transplantation. There was no significant difference in survival curves between two groups (Log-rank p = 0.53).

For further validation of the association between previous history of atopic/allergic disease and the risk of ACR, we examined 76 patients from a cohort of previous study who underwent heart transplantation at our hospital between August 2007 and May 2017 (14). Six patients (7.9%) had a history of atopic/allergic diseases such as bronchial asthma (n = 4) and atopic dermatitis (n = 3), one patient had both atopic dermatitis and bronchial asthma (**Table 1**). The basic characteristics are presented in **Table 1**. The percentage of atopic/allergic disease was slightly low possibly owing to the selection of candidates for HTx. During the chronic phase after HTx (1–3 years), patients with atopic/allergic disease tended to have a significantly higher frequency of moderate rejection [(Grade 2R (ISHLT 2004)/3A (ISHLT 1990) or higher)] than patients without atopic/allergic disease [(p = 0.012; Fisher’s exact test), Odds ratio (95% CI) 10.73 (1.75 to 65.90)] (**Figure 2**). On the other hand, there was no significant difference in the frequency of moderate rejection [p = 0.40, odds ratio (95% CI) 2.67 (0.46 to 15.53)] less than 1 year after HTx. Based on the above, the risk of graft rejection, especially during the chronic phase, increases in atopic/allergic diseases. The survival curve was not significantly different between these two groups (**Figure 3**).

**Table 1:** Basic characteristics.

	All	With allergic disease	Without allergic disease	P value
	(n = 76)	(n = 6)	(n = 70)	
Age, years	40 (29–53)	44.5 (27.5–53.0)	39.5 (28.8–53.2)	0.80
Male	53 (69.7)	6 (100)	47 (67.1)	0.09
BMI, kg/m^2^	20.0 (17.1–23.1)	23.8 (20.9–27.2)	19.8 (17.4–23.1)	0.0075
WBC, μl	5,600 (4,200–6,850)	5,400 (3,950–10,200)	5,600 (4,200–6,750)	0.79
Eosinophils,/μl	11.2 (0–51.7)	17.3 (6.3–61.5)	10.8 (0–51.9)	0.65
Hb, g/dl	11.4 (10.4–12.8)	12.1 (10.9–13.8)	11.4 (10.2–12.8)	0.20
Plt, ×10^4^/μl	21.8 (19.0–24.7)	22.0 (15.9–23.8)	21.8 (19.1–24.8)	0.54
eGFR,	51.6 (39.2–70.1)	45.9 (40.4–62.6)	52.4 (38.9–71.0)	0.59
ml/min/1.73m^2^				
CRP, mg/dl	0.06 (0.02–0.2)	0.035 (0.018–0.18)	0.06 (0.02–0.25)	0.37
BNP, pg/ml	79.2 (44.0–116.5)	64.7 (44.8–88.8)	81.5 (43.7–121.9)	0.45

All variables presented as median (interquartile range) or n (%).

Age was the age at the time of the heart transplantation. Blood test data was data one year after the heart transplantation. P-values were calculated by Fisher’s exact test, t-test or Wilcoxon rank-sum test comparing those with allergic disease and without allergic disease.

BMI, body mass index; WBC, white blood cell; Hb, hemoglobin; Plt, platelet; eGFR, estimated glomerular filtration rate; CRP, C-reactive protein; BNP, B-type natriuretic peptide.

Another lingering question is that despite immunosuppressive therapy, why did the patient develop exacerbation of atopic dermatitis? Both cyclosporin and MMF have been reported to be highly effective for atopic dermatitis. However, some studies have reported paradoxical development of atopic dermatitis after solid organ transplantation (15). Ozdemir et al. demonstrated newly-developed allergies after HTx (16). In addition, some studies have reported the development of allergies after receiving immunosuppressive therapy, but there had been no report about the mechanism of action, which should be elucidated more robustly in the future.

In fact, at our institution, prednisolone is turned off within 1 year after HTx (17). If there is concern about exacerbation of rejection due to exacerbation of atopic/allergic disease as in this case, a regimen such as continuing a small amount of steroid, which will be more effective for atopic/allergic disease, might be better.

Generally speaking, late-onset ACR has been known to have more adverse clinical outcomes as compared to early-onset ACR. However, triggers for late-onset ACR have not been identified. Future studies may focus on finding out the specific causes for late-onset ACR, which can ultimately lead to improvements in the treatment for post-HTx patients.

In a limitation of this study, the chronological association between the graft rejection and the exacerbation of atopic dermatitis in this case was difficult to presume. However, it is more likely that the state of atopic dermatitis affected the state of graft rejection because the impact derived from atopic dermatitis on the systemic immune response might be greater than the impact derived from graft rejection (18). In addition, more concise evaluation of the state of atopic dermatitis might help the correct clarification of the association between atopic dermatitis and graft rejection.

Similar to this case, no reports of graft rejection due to exacerbation of atopic dermatitis have yet to be reported. Patients with a history of allergic disorders such as atopic dermatitis may be at an increased risk of developing immunological rejection after transplantation, requiring a more intensive immunosuppressive regimen and careful follow-up.

## Conclusions

ACR following exacerbation of atopic dermatitis 3 years post-HTx is rare and has never been reported. This case highlights the importance of considering all factors that may contribute to graft rejection, regardless of diagnostic findings. However, it is also necessary to determine what processes are involved in this relationship.

## Data Availability Statement

The raw data supporting the conclusions of this article will be made available by the authors, without undue reservation.

## Ethics Statement

The studies involving human participants were reviewed and approved by the institutional review board at the University of Tokyo (approval number: 2,650). The patients/participants provided their written informed consent to participate in this study.

## Author Contributions

NK: Data collection, statistical analysis, and writing of the manuscript. EA: Conception of the idea, data collection, critical feedback on the manuscript, and writing of the manuscript. CB, MT, JI, MH, KN, KF, and HM: Data collection and critical feedback on the manuscript. MH, MO, and IK: Critical feedback on the manuscript. All authors contributed to the article and approved the submitted version.

## Funding

This work was supported by the Ministry of Education, Culture, Sports, Science and Technology of Japan through Grant-in-Aid 17K09488 (to EA).

## Conflict of Interest

EA and MH belong to the Department of Therapeutic Strategy for Heart Failure, Graduate School of Medicine, University of Tokyo, which is endowed by Actelion Pharmaceuticals Japan Ltd., Otsuka Pharmaceutical, NIPRO CORPORATION, Terumo Corp., Senko Medical Instrument Mfg., Century Medical Inc., Kinetic Concepts Inc., and St. Jude Medical.

The remaining authors declare that the research was conducted in the absence of any commercial or financial relationships that could be construed as a potential conflict of interest.
